# Insights into the pathogenesis of multiple system atrophy: focus on glial cytoplasmic inclusions

**DOI:** 10.1186/s40035-020-0185-5

**Published:** 2020-02-17

**Authors:** Seiji Kaji, Takakuni Maki, Tomoyuki Ishimoto, Hodaka Yamakado, Ryosuke Takahashi

**Affiliations:** Department of Neurology, Graduate School of Medicine, Kyoto University, 54 Shogoin-Kawahara-cho, Sakyo-ku, Kyoto, Japan

**Keywords:** Multiple system atrophy, α-Synuclein, Glial cytoplasmic inclusion, Prion, Neurodegeneration, Oligodendrocyte, Microglia, Astrocyte, Oligodendrocyte precursor cell

## Abstract

Multiple system atrophy (MSA) is a debilitating and fatal neurodegenerative disorder. The disease severity warrants urgent development of disease-modifying therapy, but the disease pathogenesis is still enigmatic. Neurodegeneration in MSA brains is preceded by the emergence of glial cytoplasmic inclusions (GCIs), which are insoluble α-synuclein accumulations within oligodendrocytes (OLGs). Thus, preventive strategies against GCI formation may suppress disease progression. However, although numerous studies have tried to elucidate the molecular pathogenesis of GCI formation, difficulty remains in understanding the pathological interaction between the two pivotal aspects of GCIs; α-synuclein and OLGs. The difficulty originates from several enigmas: 1) what triggers the initial generation and possible propagation of pathogenic α-synuclein species? 2) what contributes to OLG-specific accumulation of α-synuclein, which is abundantly expressed in neurons but not in OLGs? and 3) how are OLGs and other glial cells affected and contribute to neurodegeneration? The primary pathogenesis of GCIs may involve myelin dysfunction and dyshomeostasis of the oligodendroglial cellular environment such as autophagy and iron metabolism. We have previously reported that oligodendrocyte precursor cells are more prone to develop intracellular inclusions in the presence of extracellular fibrillary α-synuclein. This finding implies a possibility that the propagation of GCI pathology in MSA brains is mediated through the internalization of pathological α-synuclein into oligodendrocyte precursor cells. In this review, in order to discuss the pathogenesis of GCIs, we will focus on the composition of neuronal and oligodendroglial inclusions in synucleinopathies. Furthermore, we will introduce some hypotheses on how α-synuclein pathology spreads among OLGs in MSA brains, in the light of our data from the experiments with primary oligodendrocyte lineage cell culture. While various reports have focused on the mysterious source of α-synuclein in GCIs, insights into the mechanism which regulates the uptake of pathological α-synuclein into oligodendroglial cells may yield the development of the disease-modifying therapy for MSA. The interaction between glial cells and α-synuclein is also highlighted with previous studies of post-mortem human brains, cultured cells, and animal models, which provide comprehensive insight into GCIs and the MSA pathomechanisms.

## Background

Multiple system atrophy (MSA) is a progressive neurodegenerative disorder involving multiple nervous systems. The median survival from onset is about 9 years [1]. MSA symptoms, which are mainly characterized by autonomic failure, cerebellar ataxia, and parkinsonism that poorly respond to treatment used for Parkinson’s disease (PD). Depending on the predominant clinical feature, MSA patients are classified as either MSA-P, a Parkinsonian feature-dominant type, or MSA-C, a cerebellar ataxia-dominant type [2, 3]. MSA-P is the more common phenotype in most countries except some Asian countries. This difference was also confirmed pathologically by comparisons between British and Japanese post-mortem MSA cases [4].

Glial cytoplasmic inclusions (GCIs), the diagnostic hallmark of MSA, are fibrillary structures composed of misfolded α-synuclein (α-syn) [5]. This important discovery of α-syn-immunoreactive inclusions in oligodendrocytes (OLGs) raised fundamental questions: 1) what is the primary event which triggers the generation of misfolded α-syn leading to the formation of GCIs? 2) how does α-syn accumulate in OLGs, which produce few α-syn mRNA transcripts? and 3) how much are other glial cells involved in the pathogenesis of GCIs and neurodegeneration? In terms of glial cells other than OLGs, not only microglia and astrocytes but also oligodendrocyte precursor cells (OPCs) may be a role player of deep significance, considering their potential to become OLGs even in adult CNS [6]. In this review, current insights into the pathogenesis of GCIs will be highlighted based on studies from post-mortem human cases and in vitro*/*in vivo experiments replicating MSA pathology. While important viewpoints have been stated in the previously published excellent reviews, we provide an insight into the possible involvement of OPCs, featuring their notable response to extracellularly applied misfolded α-syn [7–11].

## Main text

### Diagnosis and treatment of MSA

Current consensus criteria for the diagnosis of MSA are based on three categories; definite, probable, and possible MSA [2]. Diagnosis of definite MSA requires neuropathological findings of widespread and abundant α-syn-positive GCIs, which are concomitant with striatonigral or olivopontocerebellar neurodegeneration. Diagnosis of both probable and possible MSA requires autonomic dysfunction accompanied by parkinsonism and/or by cerebellar syndrome, which symptoms are progressive and adult (> 30 years old)-onset lacking family history.

Treatment of MSA is supportive and depends on the target symptoms ranging from parkinsonism (Levodopa), orthostatic hypotension (midodrine and droxidopa), urinary tract dysfunction (anticholinergic agents), constipation (laxative therapy), breathing disorders (continuous positive air pressure and tracheotomy) and dystonia (botulinum toxin injection) [12]. Although several clinical trials have been conducted, significant improvement of MSA symptoms has not been documented except for a few medications such as rotigotine and intra-arterial administration of autologous mesenchymal stem cells [13]. Considering the efficacy of mesenchymal stem cells, intrathecal administration of these cells may be beneficial and its safety profile has just been examined through phase I/II study [14]. Nevertheless, currently, there is no potent disease-modifying therapies, which emphasizes the importance of various insights into MSA pathogenesis based on post-mortem and biochemical investigations.

### GCIs and other inclusions in MSA brains

In order to understand the non-genetically triggered pathogenesis of MSA, capturing the earliest changes during MSA disease progression is a key to unravelling its pathomechanisms. The term “minimal change” MSA describes a pathological condition of MSA that is characterized by neuronal loss restricted to the substantia nigra and locus coeruleus with widespread GCIs [15]. These cases provide critical insights into the primary changes of MSA pathology because abundant GCIs throughout the brain are found even in the absence of any clinical signs or neuronal loss, possibly serving as a key regulator of MSA disease progression [16].

GCIs were initially reported as loosely packed tubular structures adjacent to oligodendroglial nuclei by electron microscopy [17]. The majority of α-syn in GCIs is phosphorylated at Ser 129. The frequency of GCIs and the severity of neuronal cell loss are significantly correlated [18]. Other MSA-associated inclusions are less frequently observed than GCIs, including neuronal cytoplasmic inclusions (NCIs), neuronal nuclear inclusions (NNIs), and glial nuclear inclusions (GNIs), all of which are labeled with an antibody against phosphorylated α-syn and silver impregnation techniques [19]. Ultrastructurally, both GCIs and NCIs consist of granule-associated filaments with diameters of approximately 25 nm, and the filaments in these inclusions are morphologically indistinguishable [20]. Although the distribution of GCIs is always more striking than that of neuronal inclusions, some regions such as the anterior cingulate cortex and agranular frontal cortex seem to develop neuronal inclusions very frequently [21]. In MSA brains, the frequency of GCIs correlates with the severity of neuronal loss, although the possibility still exists that NCIs play a significant role in neurodegeneration, especially in the pons [18].

### Difference between GCIs and Lewy bodies (LBs)

Gai et al. described that GCI filaments are composed of central core fibrils coated with amorphous materials. Removal of this amorphous material allows visualization of 10-nm-sized central core fibrils that are strongly labeled with antibodies against α-syn, but not with antibodies against other proteins (αβ-crystallin, ubiquitin, and tubulins) [5]. Moreover, quantitative analysis of the protein composition of GCIs and LBs with immunomagnetic analysis showed that GCIs consist of 11.7% α-syn, 1.9% αβ-crystallin, and 2.3% 14–3-3 proteins (8.5, 2.0, and 1.5% in LBs, respectively) [22], with predominance of α-syn accumulation. These data suggest that fibrillary α-syn is the main component of GCIs and plays a pivotal role in GCI formation. Despite these differences, most GCI components such as cytoskeletal proteins, molecular chaperones, aggresomal proteins, and apoptosis mediators are similarly observed as components of LBs (Table 1). With all these observations in mind, some constituents predominantly expressed in OLGs such as midkine, Leu-7, transferrin, and tubulin polymerization promoting protein (TPPP)/p25α may be more specifically relevant to the formation of GCIs [95, 96].

**Table 1:** Comparison of molecular components within GCIs, NCIs, and LBs^*^ through analysis with post-mortem human brains

Protein	GCIs	NCIs	LBs^*^	References
*Chaperons*				
α-synuclein	+	+	+	[23]
Heat shock protein 70 and 90	+	ND	+	[24–26]
DJ-1	+	ND	–	[27]
αB-Crystallin	+	–	+/−	[20, 28]
*Cytoskeletal proteins*				
α/β-tubulin	+	–	+	[29–31]
Tau (non-phosphorylated)	+/−	–	+/−	[20, 30, 32, 33]
Tau (phosphorylated)	–	ND	+/−	[33–35]
Microtubule associated protein-1	+/−	ND	+	[29, 31]
Microtubule associated protein-2	–	–	+	[29, 31, 36]
p25α/TPPP (tubulin polymerization-promoting protein)	+	+	+	[37–39]
*Ubiquitin and autophagy-related proteins*				
Ubiquitin	+	+	+	[20, 40]
SUMO-1 (small ubiquitin modifier 1)	+	ND	+/−	[41, 42]
20s proteasome subunits	+	+	–	[24, 43]
HDAC6	+	–	+	[44]
Parkin	+/−	ND	+	[45, 46]
Pael-R	–	ND	+	[45]
Dorfin	+	+/−	+	[47, 48]
NEDD-8	+	+	+	[49]
NUB1 (Negative regulator of ubiquitin-like protein 1)	+	+	+	[50, 51]
Synphilin-1	+	+/−	+	[52, 53]
F-box only protein (FBXO7)	+	ND	+	[54]
p62/SQSTM1	+	ND	+	[35]
LC3	+	–	+	[55, 56]
NBR1	+	–	+	[57]
AMBRA1	+	+/−	+	[58, 59]
*Apoptosis regulators*				
Bcl-2	+	ND	ND	[60]
HtrA2/Omi	+	+	+	[61]
Parkin co-regulated gene (PACBG)	+	+/−	+	[62]
XIAP (X-linked inhibitor of apoptosis protein)	+	+	+	[63, 64]
Apoptosome (cytochrome c, Apaf-1, caspase-9)	+	+	+	[65, 66]
*Signal transduction*				
14–3-3 protein	+	+	+	[67, 68]
Mitogen-activated protein kinase (MAPK)	+	ND	ND	[69]
LRRK2	+	ND	+/−	[46, 70, 71]
*Metal-related proteins*				
Transferrin	+	ND	ND	[29]
Ferritin	+	ND	+/−	[72, 73]
Metallothionein-III	+	ND	ND	[74]
Copper/zinc superoxide dismutase	+/−	ND	+	[72, 75]
Oligodendroglial markers (proteins predominantly expressed in OLGs)^**^				
Midkine	+	–	ND	[76]
Leu-7	+	ND	ND	[77]
*Others*				
Elk1	+	ND	+	[78, 79]
cdk-5	+	ND	+	[69, 80]
P39	+	ND	ND	[81]
DARPP32	+	ND	ND	[82]
Rab5, Rabatpin5	+	ND	+	[83, 84]
Sept4	+	ND	+	[85]
Protein disulfide isomerase (PDI)	+	ND	+	[71, 86]
Apolipoprotein E	+/−	ND	+^***^	[72, 87]
Clusterin/apolipoprotein J	+/−	–	+/−	[88]
matrix metalloproteinase-2	+	ND	ND	[89]
transactive response DNA-binding protein of 43 kDa (TDP-43)	+/−	+/−	+/−	[90, 91]
*Silver stain*				
Campbell-Switzer	+	+/−	+	[92, 93]
Bodian	+	+/−	+	[29, 92, 93]
Bielshowsky	+	+/−	+	[29, 92, 93]
Gallyas	+	+	–	[29, 92–94]

The presence/absence of each protein’s expression within GCIs and NCIs in MSA brains, and within LBs in PD brains is displayed. The lists of proteins and their profiles described above are modified from [72, 95, 96].

+, positive; +/−, partially or weakly positive; −, negative; ND, not described. ^*^, described as +, or +/− when the positivity was recognized in either brainstem-type or cortical LBs; ^**^, proteins other than iron-related proteins; ^***^, amino-terminal 17 kDa fragment of Apolipoprotein E

GCI-α-syn is composed of a relatively low amount of phosphorylated α-syn with high affinity for the monoclonal antibody Syn7015 [97]. Syn7015 preferentially recognizes a distinct α-syn species with a low capability of cross-seeding tau aggregation, which may indicate a weak interaction of GCI-α-syn with tau [98]. Tau is usually restricted to neuronal axons and is not abundantly observed in glial cells in normal conditions. These findings may suggest that α-syn aggregates in GCIs are generated in OLGs but not in neurons.

In terms of an altered posttranslational modification of α-syn, it is of noteworthy that phosphorylation of NUB1, NEDD8 ultimate buster 1, may contribute to the difference between GCIs and LBs. NUB1 is previously known to colocalize with both GCIs and LBs [50]. A recent report has disclosed that phosphorylation of NUB1 at S46 accelerates aggregate degradation, and that the phosphorylation is observed in LBs but in GCIs [51].

These profiles of protein components may reflect the pre-existing environment influencing the primary step of GCI formation, and the differences of posttranslational modification against each aggregate.

### Differences between NCIs and LBs

The recent hypothesis about the prion-like properties of GCI-α-syn has led to a few curious in vivo observations, which showed a potent seeding property of intracerebrally injected GCI-α-syn with no cell-type preference among mouse central nervous system (CNS) cells [99]. Once misfolded α-syn is taken up by OLGs and induces aggregates, these aggregates generated in oligodendroglial milieu gain GCI-like seeding characteristics, regardless of the original α-syn profile of the seed [97]. In contrast, when GCI-α-syn is taken up by neurons, the induced aggregates retain the same seeding characteristics, even in the neuronal milieu. The study also revealed that even in vitro generation of aggregates from monomeric α-syn in the presence of OLG lysate is sufficient to generate GCI-α-syn strain [97]. These observations provide critically important insight into the pathogenesis of GCIs, clarifying that OLGs are the likely sites of aggregate formation from primary seeds.

In addition, these findings emphasize the importance of post-mortem investigations about the difference between two types of neuronal inclusions: NCIs and LBs. Analogous to GCIs, NCIs, the ubiquitin-immunoreactive inclusions, are labeled with antibodies to α-syn and stained with Gallyas-Braak impregnation [94]. Investigations with immunoelectron microscopy confirmed that the ultrastructure of perinuclear NCIs resembles that of GCIs rather than that of LBs [94]. Most NCIs are immunonegative for antibodies against tau, neurofilaments, and αβ-crystallin, which are the components of LBs [94] (Table 1). Taken together, many morphological profiles seen in NCIs of MSA brains are different from LBs in PD brains, possibly reflecting different cellular milieus in which each α-syn aggregate species is originally generated.

### What happens within the OLGs of MSA brains prior to the emergence of GCIs?

**Fig. 1:**
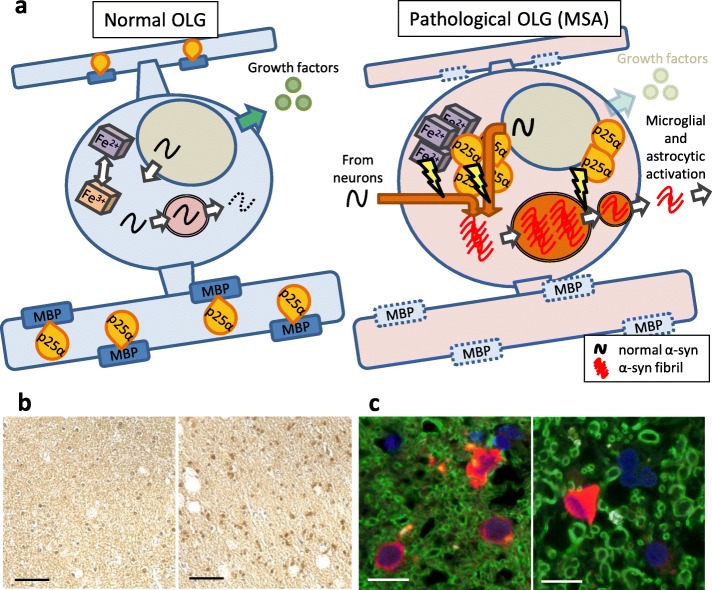
Hypothetical overview of GCI pathogenesis and post-mortem analysis of TPPP/p25α translocation. **a**: Hypothetical overview of a normal OLG (left) and a pathological OLG generating GCI (right). Left: Intracellular homeostasis is maintained by normal expression levels of myelin-associated proteins and their colocalization with TPPP/p25α as well as autophagic degradation of endogenous α-syn and balanced iron metabolism. Right: Aggregation formation is enhanced by decreased expression of myelin-associated proteins, cytosolic translocation of TPPP/p25α, impaired autophagy-lysosomal degradation, and oxidation of ferrous to ferric ions. Secretion of pathological α-syn in response to insufficient degradation leads to microglial and astrocytic activation. OLG dysfunction also causes compromised neuronal support such as reduced production of neurotrophic factors. **b**: Translocation of TPPP/p25α from myelin to cell bodies in the frontal cortex white matter of a control patient (left) and an MSA patient (right). The scale bar represents 50 μm. **c**: Localization of TPPP/p25α (red) and its interaction with MBP (green) in the frontal cortex white matter of a control patient (left) and an MSA patient (right). Blue; DAPI. The scale bar represents 10 μm

The detection of widespread myelin degeneration as the initial event in MSA brains has led to the concept of primary oligodendrogliopathy, suggesting that the pathogenesis of MSA is regulated primarily by oligodendroglial dysfunction (Fig. 1a) [17, 100, 101]. Supporting evidence shows that about 50% of non-α-syn-expressing OLGs in pontine fiber tracts of MSA brains have abnormal accumulation of TPPP/p25α and are enlarged in cell size (Fig. 1b) [37]. TPPP/p25α is an oligodendroglial-specific phosphoprotein and colocalizes with myelin basic protein (MBP) in normal human brains, and this colocalization is lost in MSA (Fig. 1c) [37]. The pathological role of TPPP/p25α was also highlighted with in vitro experiments. TPPP/p25α stimulates the polymerization of α-syn, and that both TPPP/p25α and α-syn are components of GCIs [102]. Moreover, TPPP/p25α overexpression in differentiated PC12 cells interferes with autophagic degradation of α-syn by preventing the fusion of autophagosomes with lysosomes and enhances the secretion of α-syn into the medium [103]. Although these data suggest the possibility that TPPP/p25α regulates disease onset, direct evidence for involvement of TPPP/p25α specifically in MSA pathogenesis is still insufficient. In fact, oligodendroglial TPPP/p25α accumulation is not specific to MSA, but is also detected in the brains of a newly documented tauopathy called globular glial tauopathy (GGT), in which four-repeat tau-immunoreactive inclusions develop in OLGs and astrocytes [104].

Increasing numbers of investigations about autophagy dysregulation in MSA pathology have been published in recent years. This trend may have been accelerated by the notion of neuronal inclusion formation in autophagy-deficient *Atg5*-knockout mice, as well as by the disclosure of the genetic association between *GBA* variants and incidence of MSA in the context of autophagic dysregulation [3, 105]. Indeed, in vitro observations have revealed that pharmacologic and genetic inhibition of autophagy causes significant accumulation of both endogenous and exogenously applied α-syn in oligodendroglial cells [11, 106]. Dysfunction of the autophagy-lysosome system in MSA is also regulated by transcriptional and epigenetic mechanisms. A recent report showed that the autophagy-suppressing microRNAs, miR-101 and let-7b, are significantly increased in the striatum of MSA brains [107]. The report also clarified that lentiviral delivery of an anti-miR-101 construct to the striatum of the MBP-α-syn transgenic mouse model of MSA results in reduced oligodendroglial α-syn accumulation and improved autophagic clearance. Another post-mortem study showed that GCIs contain an upstream protein of autophagy, autophagy/beclin1 regulator 1 (AMBRA1), the overexpression of which leads to mild reduction of abnormal α-syn in HEK293 cells co-transfected with S129E α-syn [58]. Moreover, the protein expression levels of AMBRA1 are increased in MSA brains, whereas those of an upstream regulator, TNF receptor associated factor 6, are significantly decreased, suggesting that the upstream autophagy regulation pathways are impaired [58]. Nevertheless, evidence is still lacking regarding whether autophagic dysregulation precedes α-syn accumulation in OLGs. Interestingly, induced pluripotent stem cell (iPSC)-derived dopaminergic neurons from MSA patients show aberrant autophagic machinery without obvious inclusion formation [108]. These findings suggest the presence of a general autophagy defect in MSA brains as a prodromal condition predisposing the individual to the emergence of GCIs.

Iron accumulation in affected areas of MSA brains is a pathological hallmark of the disease [109]. In addition to the increase in the total iron concentration, expression of the iron storage protein, ferritin, is increased, and the iron export protein, ferroportin, is decreased in the pons of patients with MSA, suggesting the presence of dysregulated bioavailability of iron in MSA brains [110]. OLGs play critical roles in iron homeostasis, as these cells contain iron and express iron-binding proteins such as transferrin in the normal CNS [111]. Iron levels in basal ganglia (putamen, globus pallidus, and caudate nucleus) are physiologically higher than those of the other brain areas [112]. These physiological profiles of iron expression may be partially associated with the predisposition of iron accumulation in OLGs and basal ganglia of MSA brains. The presence of an iron responsive element in the 5′-untranslated region of the α-syn transcript implies the potential for induction of excessive α-syn production triggered by iron accumulation [113]. In vitro experiments have clarified that various metals including iron cause significant acceleration in the rate of α-syn fibril formation, and both ferritin and transferrin are contained within GCIs [29, 114]. Although these findings suggest that iron dysregulation may be a potential predisposing event of MSA pathogenesis, the alteration in the iron concentration has not been documented in the locus coeruleus of MSA brains where severe neuronal loss is frequently observed [115]. Moreover, a MRI study shows that putaminal iron accumulation occurs under volume atrophy or change in microstructural integrity, implying that putaminal iron deposition in MSA brains is a secondary byproduct of neurodegeneration [116]. At present, whether iron dysregulation is a primary event underlying the pathogenesis of GCIs or a secondary event as the result of neuronal degeneration and subsequent microglial activation is unclear [117].

### How GCI pathology spreads?

The concept of α-syn as a prion-like protein originates from the description of LBs in grafted neurons within the post-mortem brains of PD patients who were transplanted with human fetal mesencephalic dopaminergic neurons [118, 119]. Further in vitro studies have proven that extracellularly applied α-syn fibrils induce endogenous soluble α-syn in primary neurons to form insoluble fibrillary α-syn aggregates [120]. The prion-like property of α-syn was also confirmed with GCI-α-syn. Injection of MSA brain homogenates into α-syn transgenic mice revealed a more potent and prion-like property of GCI-α-syn compared with that of LB-α-syn [97, 99]. The prion-like property of GCI-α-syn persists even after serial propagation in α-syn transgenic mice [121]. These findings led to the hypothesis that prion-like propagation of GCI-α-syn pathology contributes to the disease progression of MSA. Interestingly, GCIs are immunoreactive to Rab5, a Rab protein that is involved in the transport of cell surface molecules to early endosomes [83]. Rab5 proteins are expressed not only in neurons but also in OLGs, and their expression increases with OLG differentiation [122, 123]. The colocalization of fibrillary α-syn and early endosomes may be proof of cell-to-cell transfer by which α-syn pathology spreads in MSA brains. Nevertheless, trans-synaptic propagation of α-syn pathology, which is suggested in PD pathology, cannot be simply applied to the OLG-specific distribution of α-syn pathology in MSA [124, 125]. In fact, GCIs seem to be distributed randomly or present in clusters, and their spatial patterns are different from those of neuronal inclusions in other neurodegenerative diseases [126]. Considering these observations, cell-to-cell propagation of GCI-α-syn pathology may occur in a distinctive pattern.

Identifying the route and regulator of OLG-specific disease propagation in MSA pathology is challenging. Although some ELISA-based studies did not detect a significant change in total α-syn concentrations in the cerebrospinal fluid (CSF) of MSA patients, an increase in phosphorylated oligomeric α-syn may be present [127, 128]. However, α-syn-immunoreactive inclusions in mouse brains injected with GCI-α-syn are observed predominantly within neurons, rather than within OLGs [97, 99]. These observations may indicate the existence of exclusive OLG-to-OLG communications specific to MSA brains.

Overall, some unanswered questions remain regarding the pathological contribution of the prion-like property of GCI-α-syn for MSA disease progression. Even if the prion-like property of GCI-α-syn explains how pathological conformation spreads from cell to cell in MSA brains, the explanation of what triggers the emergence of the primary seeds is still missing.

### Future hot topics: the possible involvement of OPCs in the spreading of GCI pathology

To further our understanding of the mechanism underlying the propagation of GCI pathology, two aspects of the GCI development require scrutiny: 1) How does misfolded α-syn induce prion-like propagation between OLGs? and 2) Which cell produces the excessive amount of α-syn, serving as the main source of aggregated α-syn in GCIs?

Unlike neurons, OLGs possess a unique repair mechanism, which is enabled by the presence of OPCs. OPCs are abundant in adult brains, making up 5–8% of the glial cell population [6]. In response to various types of CNS damage, OPCs show extensive proliferation, migration, and differentiation in an attempt to compensate for demyelination [129, 130]. A few investigations suggest that the numbers of OPCs are increased in MSA brains [131]. However, the number of mature OLGs is reported to be not decreased, even in the presence of abundant GCIs, neuronal loss, and myelin loss [101, 132, 133]. The pathological finding of myelin loss accompanied by preserved numbers of OLGs in MSA brains may imply that α-syn-induced impairment of remyelination involves reduced myelin turnover and defective replacement of damaged OLGs [101].

**Fig. 2:**
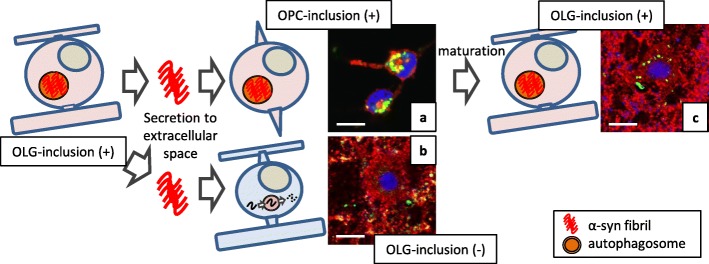
Difference in the response to extracellularly applied pathological α-syn between two oligodendroglial cells. Pathological α-syn with seeding property hypothetically propagates from OLG to OLG. Extracellular α-syn fibrils do not induce inclusions when they are applied to mature OLGs. Seed internalization can occur more drastically during the immature state of OLG differentiation including the precursor state. The mechanism which regulates the uptake of misfolded α-syn may be shared by normal OPCs and pathological OLGs in MSA brains, but not by normal OLGs. **a**: intercellular localization of α-syn (green)-immunoreactive inclusions in platelet-derived growth factor receptor α (red)-positive primary rat OPCs, which were incubated for 72 h with 1 μM human recombinant α-syn pre-formed fibrils (PFFs). **b**: MBP (red)-positive primary rat OLGs with extracellular Thioflavin S (green) immunoreactivity, which were incubated for 24 h with 1 μM human recombinant α-syn PFFs after maturation. **c**: MBP (red)-positive primary rat OLGs containing Thioflavin S (green)-positive intracellular inclusions. OLGs were differentiated from OPCs that were pre-incubated for 24 h with 1 μM human recombinant α-syn PFFs. **a**-**c**: Each scale bar represents 10 μm. Blue; DAPI.

**Fig. 3:**
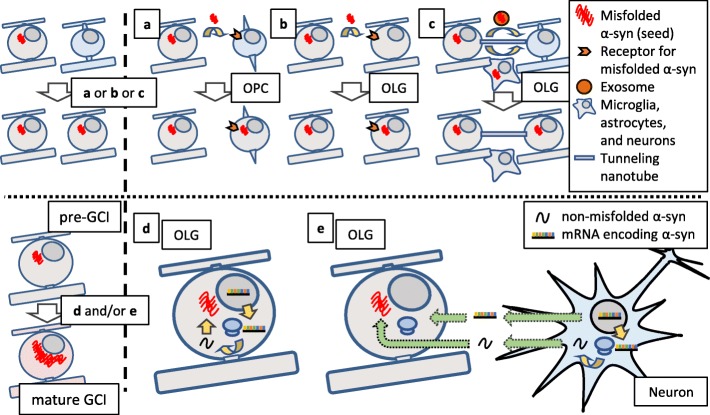
Hypothetical schema showing OLG-to-OLG propagation and accumulation of pathological α-syn. Pathological α-syn with seeding property hypothetically propagates from OLG to OLG leading to the spreading of GCI pathology. Given that the uptake of extracellularly-applied misfolded α-syn is not usually observed in normal OLGs, the entry of misfolded α-syn into oligodendroglial cells is mediated through an unidentified mechanism (**a**-**c**). Once the misfolded α-syn (pre-GCI) enters oligodendroglial cells, the pre-GCI self-assembles through the interaction with non-misfolded α-syn, resulting in the formation of perinuclear fibrillary structure (mature GCIs). The non-misfolded α-syn may be derived from OLGs or neurons (**d**, **e**)

An ongoing debate remains regarding whether OPCs are involved in MSA pathology. The difficulty in the immunohistochemical detection of OPCs in human tissues is probably due to the excessive vulnerability to fixation and the low specificity of antigens which are enriched in OPCs [134]. However, it seems very likely that some immature oligodendrocytes in MSA brains contain α-syn-immunoreactive inclusions [11, 131]. In vitro experiments using primary culture indicate that extracellularly applied α-syn is taken up by OPCs and disrupts their maturation [11]. Importantly, although recombinant human α-syn pre-formed fibrils (PFFs), which induce inclusions in neurons, are incorporated into OPCs leading to intracellular inclusion formation, α-syn PFFs do not induce inclusions when applied to OLGs (Fig. 2a and b) [11, 120]. Furthermore, once α-syn PFFs are incorporated into OPCs, intracellular inclusions can be detected even after their maturation, causing insufficient neuro-supportive function (Fig. 2c) [11]. The presence of unique resistance against inclusion formation in mature OLGs is also speculated from in vivo observations using mice injected with brain extracts from MSA patients [99]. Given the fact that normal OLGs cannot take up extracellular seeds, three hypothetical explanations have been proposed for seed propagation between OLGs in MSA brains: 1) the uptake of extracellular seeds occurs before complete OPC maturation (Fig. 3a), 2) OLGs in MSA acquire an abnormal uptake mechanism that allows the invasion of extracellular seeds (Fig. 3b), and 3) seeds are transferred to OLGs via tunneling nanotubes or extracellular vehicles such as exosomes and other glial cells (microglia and astrocytes) [135–137] (Fig. 3c).

At present, the uptake mechanism of α-syn PFFs into OPCs is not clarified. Considering the possibility that normal OPCs and OLGs in MSA brains share the same mechanism of α-syn PFFs uptake (Fig. 3c), it is critically important to elucidate the regulators of their uptake in OPCs. Inhibitors of clathrin-mediated endocytosis did not affect α-syn PFFs uptake into OPCs, although RNA-seq analysis of α-syn PFF-treated OPCs showed increased gene expressions of Rab proteins which mediate endocytosis [11]. The quantification of gene and protein expression levels of lymphocyte activation gene-3 (LAG3), which is a membranous protein known to bind specifically with α-syn PFFs, also failed to specify how α-syn PFFs uptake into OPCs is controlled [11, 138]. Thorough investigations of oligodendroglial transmembrane proteins and their association with pathological α-syn may reveal the mechanism of cell-to-cell propagation of the pathological seeds.

It is also of note that disclosure of OPC pathology in MSA brains may contribute to the development of cell therapies. In fact, OPC transplantation successfully promoted remyelination and functional recovery in a chronic demyelinated mouse model [139]. Transplantation of OPCs, which are genetically modified to resist α-syn pathology, may not only encourage the replacement of the impaired OLGs but also arrest disease progression in MSA brains.

### How α-syn accumulates in OLGs: the source of α-syn in GCIs

The internalized misfolded α-syn (Fig. 3 left, pre-GCI) presumably self-assembles through the interaction with a large amount of α-syn to eventually form the perinuclear fibrillary structure (Fig. 3 left, mature GCI). There are two possibilities regarding which cell produces the majority of α-syn composing GCIs in MSA brains: OLGs and neurons (Fig. 3d, e).

OLGs express less α-syn protein than neurons. Previous assays with in situ hybridization were not sufficiently sensitive to detect α-syn mRNA within OLGs of either control or MSA brains [140, 141]. In contrast, analysis of oligodendroglial mRNA expression using laser-capture microdissection showed a 1.6-fold increase in *SNCA* mRNA expression in MSA OLGs compared with control OLGs; however, this increase was not statistically significant for inducing pathological aggregate formation [142]. In rat primary culture, α-syn protein expression in OLGs is approximately 30% of that in neurons [11]. iPSC-derived OLGs from healthy individuals and MSA patients showed the presence of α-syn protein expression in OLG lineage cells, although the expression is reduced by up to 70% when cells are fully mature [143]. Curiously, our recent in vivo observation of wild-type mice brains injected with aggregated α-syn revealed that the emergence of oligodendroglial α-syn pathology occurs over several months after that of neuronal α-syn pathology [144]. This notion is extremely important considering that modest amount of endogenous α-syn expression in wild-type OLGs can contribute to GCI formation over a long period.

Currently, the leading hypothesis for α-syn accumulation within OLGs is that α-syn is transferred from neurons to OLGs (Fig. 3e). Given that GCI-specific α-syn fibrils can be generated only within OLGs, the soluble form of α-syn may be transferred from neurons to OLGs [97]. A few in vitro and in vivo studies have tried to observe neuronal release and oligodendroglial uptake of α-syn [145, 146]. The neuron-to-OLG communication may be mediated not only by the delivery of proteins but also by the delivery of mRNA and microRNA [147]. In spite of these possibilities, however, the elevation of neuronal α-syn mRNA expression has not been confirmed in MSA brains [142, 148]. These facts may imply that the presence of α-syn overproduction in OLGs or neurons is less relevant to the pathomechanism of inclusion formation compared with the conformational change of α-syn [149, 150]. This notion is also supported by recent studies which revealed even minute amount of endogenous α-syn in oligodendroglial cells can contribute to inclusion formation [11, 151].

### The involvement of microglia and astrocytes in MSA pathology

Microglia and astrocytes are involved in the disease progression of MSA, which notion is supported by observation of increased numbers of these cells in MSA brains [133, 152, 153]. Activation of microglia seems evident in white matter regions where α-syn inclusions are abundantly observed [154]. Evidence is still lacking with regard to whether microglial activation precedes the emergence of GCIs or prodromal symptoms such as rapid eye-movement sleep behavior disorder [155]. Microglial activation due to fibrillary α-syn and subsequent production of pro-inflammatory cytokines such as interleukin (IL)-1β through NACHT, LRP and PYD domain-containing protein 3 (NLRP3) and Apoptosis-associated speck-like protein containing a CARD (ASC) inflammasome activation is closely linked to dopaminergic neurodegeneration in MSA brains [152, 156]. In addition, some in vitro studies have demonstrated that misfolded α-syn is responsible for microglial activation via signaling through Toll-like receptors [157, 158].

Reactive microglia in α-syn pathology may also serve as a potential vehicle for cell-to-cell α-syn spread. This notion is supported by the presence of microglial cells bearing α-syn inclusions distal from GCIs in MSA brains [137]. Microglia take up exosomes released by α-syn-containing OLGs via macropinocytosis, but have a low capability of degrading fibrillar α-syn in vitro [159]. Given the crucial roles of the NLRP3-ASC inflammasome in amyloid β (Aβ)- and tau-induced microglia on accumulation and propagation of Aβ and tau [160, 161], reactive microglia with a high migratory capacity in α-syn pathology may also accelerate development of α-syn aggregates and spread via uptake and re-release of α-syn. Further studies are needed to examine if blocking the microglial inflammasome, α-syn uptake, or migration could indeed mitigate disease progression in MSA animal models.

Astrocytes also appear to be activated by α-syn in MSA brains [162]. Accumulation of α-syn inclusions in astrocytes can be observed among subpial and periventricular regions of MSA patients, especially those with a long disease duration [163]. Emergence of α-syn inclusions is also reported with primary astrocyte culture, which is exposed to synthetic and patient-derived aggregated α-syn [164, 165]. It is, however, still unclear whether these astrocytic responses enhance neurodegeneration or neuroprotection in MSA brains. On one hand, reactive astrocytes, which take up α-syn, secrete increased levels of cytokines (IL-1α, IL-1β, IL-6, etc.), colony-stimulating factors, and chemokines, triggering inflammatory neurodegenerative processes [166]. On the other hand, the accumulation of α-syn within astrocytes may reflect the process of astrocytic degradation, which is neuroprotective against cytotoxic α-syn [164]. Another possible mechanism of astrocyte-mediated neuroinflammation may be mediated through the conversion of normal astrocytes into the neurotoxic A1 phenotype through α-syn-induced microglial activation. Notably, a recent study reported that the glucagon-like peptide-1 agonist, NYL01, which blocks microglial activation and the generation of A1 astrocytes, may prolong the survival and reduce the neuropathology in a model of α-synucleinopathy [167]. In terms of non-cell autonomous α-syn spread, PD patient-specific iPSC-derived dysfunctional astrocytes accumulate and transfer pathological α-syn species to healthy dopaminergic neurons, resulting in neurodegeneration [168].

### From GCI to neurodegeneration: how can this process be prevented?

Although various players are suspected to contribute to neurodegeneration in MSA, the main contributing factor remains elusive. One important finding from post-mortem analysis is the unique cell populations in MSA brains: neuronal loss with preserved numbers of OLGs and increased numbers of microglia [101, 131–133]. Based on these observations, α-syn-induced neuronal loss in MSA can be attributed to three possible components: 1) cytotoxicity of abnormal α-syn against neurons, 2) insufficient neuronal support from OLGs, and 3) cytotoxicity mediated through microglial and astrocytic activation.

NCIs are commonly observed in MSA brains, and this observation suggests the presence of direct interactions between α-syn and neurons [21]. Some oligomeric α-syn species exert their neurotoxicity through induction of calcium ion flux [169]. CSF from MSA patients induces cytotoxicity via activation of endoplasmic reticulum stress and autophagy in cultured neuroblastoma cells and the substantia nigra of CSF-injected mice [170]. This cytotoxicity may be mediated through the uptake of soluble oligomeric α-syn species.

Reduced expression of myelin-associated proteins and neurotrophic factors, such as brain-derived neurotrophic factor (BDNF) and glial-derived neurotrophic factor (GDNF), was reported in an MSA mouse model [101, 131, 171]. In experiments with primary rat OLG culture, OLGs show specifically high mRNA expression of GDNF, and conditioned medium from OLGs has a strikingly positive effect on the survivability of primary neurons [11]. Yet the impaired neurotrophic support from OLGs has not been fully evaluated on a cellular level in MSA brains.

### MSA animal models: how useful?

The main approaches to replicating MSA pathology in animals include administration of neurotoxins and generation of transgenic animals. Intracerebral injection of 6-hydroxydopamine and quinolinic acid and systemic administration of 1-methyl-4-phenyl-1,2,3,6-tetrahydropyridine, rotenone, and 3-nitropropionic acid are commonly used [172]. With the objective of replicating GCIs, the pathological hallmark of MSA, transgenic overexpression of α-syn under control of the promoter of OLG markers is often used. Each transgenic model using the promotor of 2′,3′-cyclic nucleotide 3′-phosphodiesterase (CNP), myelin basic protein (MBP), and proteolipid protein (PLP) has slightly different characteristics, although all models develop insoluble aggregates containing phosphorylated α-syn within OLGs (Table 2). Moreover, in all of these models, neuronal degeneration and predominant oligodendroglial α-syn aggregate formation were demonstrated, consistent with the non-cell autonomous mode of neurodegeneration in MSA [173, 175, 177]. The limitations applicable to all types of models are as follows: 1) high expression levels of oligodendroglial α-syn mRNA/protein are not consistent with MSA brain pathology [148], and 2) none of these animal models replicates olivopontocerebellar pathology of MSA brains (Table 2).

**Table 2:** Comparison of human α-syn overexpression mouse models

Promotor	CNP	MBP^*^	PLP
Phosphorylated α-syn aggregates in OLGs	++	++	++
Neuronal loss			
Striatonigral system	–	++^**^	++
Olivopontocerebellar system	ND	ND	–
Spinal cord	+	–	++^***^
Demyelination	+	++	–
Microglial activation	ND	++	++
Phenotype			
Motor	++	++	+
Non-motor	–	ND	++
Reference	[173, 174]	[175, 176]	[177, 178]

The results of immunohistochemical and phenotypic analysis of each MSA mouse model are highlighted.

++, present within 12 months of age; +, present after 12 months of age; −, not clearly observed; ND, not described. ^*^, moderate expresser line; ^**^, not significant in substantia nigra; ^***^, mainly parasympathetic outflow

It is also of note that injection of synthetic α-syn PFF into the brains of non-α-syn-overexpressing wild-type mice enables replication of oligodendroglial α-syn pathology after long post-injection intervals of several months [144]. Further approaches for the development of non-α-syn-overexpression MSA models may contribute to the interpretation of disease pathogenesis, which precedes the accumulation of pathological α-syn in OLGs.

## Conclusions

As recently described, the GCI-α-syn species can maintain its conformational and prion-like property even in a different cellular environment [97]. Although direct evidence is still lacking with regard to whether prion-like propagation contributes to the disease progression of MSA, further understanding of α-syn conformation and the components of pathological inclusions may provide critical information about the pathogenesis.

Given that GCIs precede neuronal loss in MSA brains and misfolded α-syn exerts neurotoxicity, prevention of the generation and propagation of GCIs seems to modify the disease progression of MSA. As for the primary generation of GCIs, even though age-dependent decline of OLG function may precede triggering the misfolding of α-syn in MSA, GCIs have not been replicated in vivo without overexpressing oligoderndroglial α-syn [7]. While a few recent studies have described the role of endogenous α-syn in GCI formation, the mechanism of misfolded α-syn uptake into oligodendroglial cells needs to be elucidated in order to block the OLG-to-OLG propagation of GCIs [11, 151].

Importantly, our recent observations have shown that OPCs incorporate extracellularly applied fibrillar α-syn, whereas their maturation results in decreased uptake activity [11]. Although not only OPCs but also other glial cells can take up misfolded α-syn, the emergence of α-syn-immunoreactive inclusions in OPCs is of great pathological significance, considering their capability to become OLGs [10]. Disclosure of the uptake mechanism, which regulates the entry of misfolded α-syn into oligodendroglial cells probably contributes to the development of disease-modifying therapy against MSA.

## Data Availability

All data generated or analyzed during this study are included in this published article.

## Abbreviations

AMBRA1: Autophagy/beclin1 regulator 1
BDNF: Brain-derived neurotrophic factor
CNP: 2′,3′-cyclic nucleotide 3′-phosphodiesterase
CNS: Central nervous system
*COQ2*: Coenzyme Q2
CSF: Cerebrospinal fluid
DARPP32: Dopamine- and cAMP-regulated neuronal phosphoprotein
FBXO7: F-box only protein 7
*GBA*: glucocerebrosidase
GCI: Glial cytoplasmic inclusion
GDNF: Glial-derived neurotrophic factor
GNI: Glial nuclear inclusion
HDAC6: Histone deacetylase 6
IL: Interleukin
iPSC: Induced pluripotent stem cell
LB: Lewy body
LRRK2: Leucine-rich repeat kinase 2
MAPK: Mitogen-activated protein kinase
MBP: Myelin basic protein
MSA: Multiple system atrophy
NBR1: Next to BRCA1 gene 1 protein
NCI: Neuronal cytoplasmic inclusion
NEDD8: Neural precursor cell expressed developmentally down-regulated protein 8
NLRP3: NLR family, pyrin domain containing 3
NNI: Neuronal nuclear inclusion
NUB1: Negative regulator of ubiquitin-like protein 1
OLG: Oligodendrocyte
OPC: Oligodendrocyte precursor cell
PACBG: Parkin co-regulated gene
PARP-1: Poly (adenosine 5′-diphosphate-ribose) polymerase-1
PD: Parkinson’s disease
PDI: Protein disulfide isomerase
PFF: Pre-formed fibril
PLP: Proteolipid protein
Ser: Serine
*SNCA*: α-synuclein
SUMO-1: Small ubiquitin modifier 1
TDP-43: Transactive response DNA-binding protein of 43 kDa
TPPP: Tubulin polymerization promoting protein
*TREM2*: Triggering receptor expressed on myeloid cells 2
XIAP: X-linked inhibitor of apoptosis protein
α1BAR: α1B adrenergic receptor
α-syn: α-synuclein
